# Diagnostic and prognostic significance of tartrate‐resistant acid phosphatase type 5b in newly diagnosed prostate cancer with bone metastasis: A real‐world multi‐institutional study

**DOI:** 10.1111/iju.15063

**Published:** 2022-10-28

**Authors:** Gaku Yamamichi, Taigo Kato, Satoru Yumiba, Eisuke Tomiyama, Yoko Koh, Kosuke Nakano, Makoto Matsushita, Yujiro Hayashi, Yu Ishizuya, Tadashi Watabe, Koji Hatano, Atsunari Kawashima, Takeshi Ujike, Yutaka Ono, Tsuyoshi Takada, Shingo Takada, Ryoichi Imamura, Norio Nonomura, Motohide Uemura

**Affiliations:** ^1^ Department of Urology Osaka University Graduate School of Medicine Osaka Japan; ^2^ Department of Urology Osaka Police Hospital Osaka Japan; ^3^ Department of Urology Higashiosaka City Medical Center Osaka Japan; ^4^ Department of Nuclear Medicine and Tracer Kinetics, Graduate School of Medicine Osaka University Osaka Japan; ^5^ Department of Urology Minoh City Hospital Osaka Japan

**Keywords:** bone metastasis, bone turnover marker, cancer‐specific survival, diagnosis, prostate cancer

## Abstract

**Objectives:**

Approximately, 90% of men with advanced prostate cancer will develop bone metastasis. However, there have been few reports about noninvasive biomarker to detect and predict clinical outcome of bone metastasis (BM) in prostate cancer patients.

**Methods:**

We examined 1127 patients who underwent prostate biopsy from August 2012 to June 2017. We also investigated bone turnover markers such as bone‐specific alkaline phosphatase, type I collagen cross‐linked N‐terminal telopeptide, C‐terminal pyridinoline cross‐linked telopeptide of type I collagen, and tartrate‐resistant acid phosphatase type 5b (TRACP 5b).

**Results:**

A total of 282 patients were diagnosed as prostate cancer with complete clinical data, and 34 patients with bone metastasis. Multivariate analysis revealed C‐terminal pyridinoline cross‐linked telopeptide of type I collagen, tartrate‐resistant acid phosphatase type 5b, and prostate‐specific antigen (PSA) were independent biomarkers in detection of BM (*p* < 0.05, respectively). Furthermore, we developed predictive model formula based on tartrate‐resistant acid phosphatase type 5b and PSA, for which the area under the curve was 0.95. In patients with bone metastasis, multivariate cox proportional hazards analysis revealed that this model was significantly associated with poor clinical outcome of cancer‐specific survival (*p* < 0.05). In validation cohort with 137 patients, we also confirmed the utility of this model for diagnosis of BM (the area under the curve = 0.95).

**Conclusions:**

Our developed formula of tartrate‐resistant acid phosphatase type 5b in accordance with PSA may serve as the useful tool in diagnosis and prediction of clinical outcome for prostate cancer with bone metastasis.

**Abbreviations & Acronyms**
AUC: area under the curve
BAP: bone‐specific alkaline phosphatase
BM: bone metastasis
CI: confidence interval
CRP: C‐reactive protein
CSS: cancer‐specific survival
I CTP: carboxy‐terminal pyridinoline cross‐linked telopeptide parts of type I collagen
NPV: negative predictive value
NTx: cross‐linked N‐terminal telopeptides of type I collagen
OR: odds ratio
OS: overall survival
PCa: prostate cancer
PSA: prostate‐specific antigen
ROC: receiver operating characteristic
tALP: total alkaline phosphatase
TRACP 5b: tartrate‐resistant acid phosphatase type 5b

## INTRODUCTION

In 2020, there were over 1 414 000 estimated new cases of prostate cancer (PCa) worldwide.^1^ Although localized PCa has a 5‐year survival rate approaching 100%, this rate drops to 31% for metastatic PCa.^2^ Among all types of tumors, PCa has the highest incidence of bone metastasis (BM),^3^ and prospective study with newly diagnosed PCa showed a prevalence of BM with 13.7%.^4^ Generally, BM is mainly diagnosed by ^99m^Tc‐based bone scintigraphy. However, bone scintigraphy has some disadvantages such as low specificity, high cost, and risk of radiation exposure.^5^ To overcome these limitations, it is urgent to pursue more convenient and less invasive biomarkers in clinical settings.

So far, bone turnover markers have been investigated as convenient tools in the diagnosis of BM in PCa.^6^ However, there are no unified marker due to differences in the types of systemic treatment which affects bone metabolism in cancers.^7^, ^8^ Therefore, it is important to focus on only untreated PCa patients with BM.

In this study, we revealed the model equation utilizing tartrate‐resistant acid phosphatase type 5b (TRACP 5b) of serum bone turnover markers in accordance with serum prostate‐specific antigen (PSA) as the accurate tool detecting BM in newly diagnosed PCa. In addition, we demonstrated that this model can apply to predictive tool for the presence of BM and cancer‐specific survival (CSS) in newly diagnosed PCa. Collectively, this model equation may lead to a better alternative tool to predict BM and prognosis of PCa patients.

## METHODS

### Patient selection

For the discovery cohort, we enrolled 1127 patients who underwent prostate needle biopsy and were diagnosed as PCa consecutively between August 2012 and June 2017 at Osaka University Hospital. Patients lacking laboratory data (PSA and 5 types of bone turnover markers), imaging data, or with history of other types of cancer were excluded from this study. All PCa patients received both computed tomography and ^99m^Tc‐based bone scintigraphy at initial diagnosis, which enables clinical diagnosis of BM. For cases with unclear BM diagnosis, additional local magnetic resonance imaging, systemic ^18^F‐labeled prostate‐specific membrane antigen‐1007 positron emission tomography,^9^ and bone biopsy were performed, or an additional radiological imaging test was performed within 3 months after hormone therapy to evaluate the reactivity and strictly made the diagnosis of BM. Based on these results, the number and location of BM were examined and the tumor volume was classified as low or high group according to the CHAARTED trial, with high‐volume disease defined as presence of visceral metastases and/or at least four bone lesions with at least one lesion outside of the vertebral column and/or pelvis.^10^ As the validation cohort, we evaluated PCa patients diagnosed from August 2020 to September 2021 at Osaka University Hospital, Higashiosaka City Medical Center, Osaka Police Hospital, and Minoh City Hospital. This study was approved by the Institutional Review Board of Osaka University Hospital (# 13397‐19).

### Blood samples

Serum PSA (Beckman Coulter) was measured on an outpatient and other blood samples were collected between 8:00 a.m. and 10:00 a.m. while in the hospital for prostate biopsy^11^ as described previously. As serum bone turnover markers, we measured a total of 5 types of bone turnover markers including total alkaline phosphatase (tALP), bone‐specific alkaline phosphatase (BAP), cross‐linked N‐terminal telopeptides of type I collagen (NTx), carboxy‐terminal pyridinoline cross‐linked telopeptide parts of type I collagen (I CTP), and TRACP 5b. tALP, BAP, NTx, and TRACP 5b were measured by enzyme assay with 2‐amino‐2‐methyl‐1‐propanol buffer (Shino‐Test Corporation), chemiluminescent enzyme immune assay (Beckman Coulter), enzyme‐linked immune sorbent assay (Alere Medical), and enzyme immunoassay (Nittobo Medical), respectively. We also measured I CTP with radioimmunoassay (Orion Daignostica). According to the institutional upper limit of normal, we defined renal dysfunction as serum creatinine (FUJIFILM Wako Pure Chemical Corporation) >1.2 mg/dl and hepatic dysfunction as serum aspartate aminotransferase (FUJIFILM Wako Pure Chemical Corporation) or alanine aminotransferase (FUJIFILM Wako Pure Chemical Corporation) >40 IU/L or serum total bilirubin (FUJIFILM Wako Pure Chemical Corporation) >1.2 mg/dl.

### Statistical analysis

The clinical characteristic data were examined by Mann–Whitney *U* test. Univariate and multivariate logistic regression analyses were performed to assess the relative contributions to diagnosis of BM. Stepwise multivariate logistic regression analysis was used to create the model equation, and the predicted probability of BM was estimated as *p* = 1/(1 + *e* ^−*x*^) and predictive accuracy of BM was verified by receiver operating characteristic (ROC) curves analyses to use area under the curve (AUC), as we previously reported.^11^, ^12^ CSS and overall survival (OS) were estimated using the Kaplan–Meier method and cox proportional hazard regression. All *p* values were two‐sided and differences were considered statistically significant when *p* < 0.05. Data were analyzed with JMP Pro 15 (SAS Institute Inc.).

## RESULTS

### Discovery cohort

**FIGURE 1:**
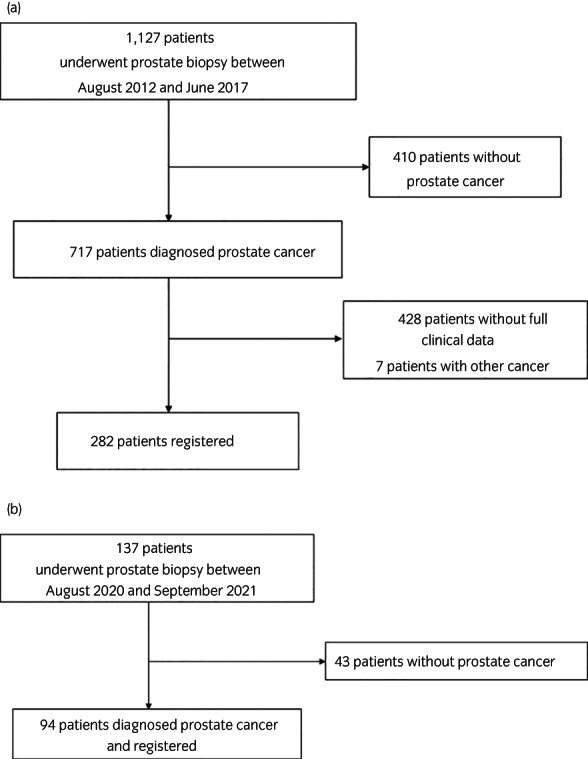
The flow chart of patient selection process in the discovery cohort (a) and validation cohort (b).

In the discovery cohort, of the 1127 patients with prostate biopsy, 282 PCa patients were included in the analysis (Figure 1a). The clinical characteristics of all patients were summarized in Table 1. In 282 PCa patients, 34 patients were identified with BM, including 7 low tumor volume and 27 high tumor volume, with the median follow‐up time of 61 months (range 1–101 months). During the observational period, 26 patients died, including 12 PCa‐related death. In the BM present group, all bone turnover markers were significantly higher when compared to those in the BM absent group (*p* < 0.01). Renal dysfunction was observed in 24 patients (8.5%), and hepatic dysfunction was observed in 40 patients (14.2%). In total, renal or hepatic impairment was observed in 61 (21.6%) of all 282 patients. The median level of each bone turnover marker was used as the cutoff value (218.5 IU/L for tALP, 12.2 μg/L for BAP, 13.6 nmol BCE/mmol Cr for NTx, 3.8 ng/ml for I CTP, 335 mIU/dl for TRACP 5b, and 9.91 ng/ml for PSA). In the multivariate analysis, I CTP, TRACP 5b, and PSA were significantly associated with the presence of BM (Table 2).

**TABLE 1:** Patients' characteristics

	Discovery cohort			Validation cohort		
	BM (+)	BM (−)	*p* value	BM (+)	BM (−)	*p* value
	(*n* = 34)	(*n* = 248)		(*n* = 28)	(*n* = 66)	
Age, median	73.5 (53–84)	70 (51–86)	0.038	77 (53–92)	73 (55–84)	0.15
ECOG‐PS						
<1	7	238	<0.01	4	47	<0.01
≧1	27	10		24	19	
Gleason score						
<8	5	160	<0.01	1	44	<0.01
≧8	29	88		27	22	
CRP (mg/dl)	0.10 (0–8.7)	0.02 (0–14.9)	<0.01	—	—	—
PSA (ng/ml)	227.5 (5.0–9436)	9.1 (1.6–281.4)	<0.01	530.1 (15.2–6840)	9.1 (3.0–499.3)	<0.01
tALP (IU/L)	319 (143–2815)	210 (106–711)	<0.01	—	—	—
BAP (μg/L)	21.3 (8.2–533)	11.9 (5.3–44.6)	<0.01	—	—	—
NTx (nmol BCE/mmol Cr)	20.4 (10.1–65.8)	13.2 (6.9–90.0)	<0.01	—	—	—
I CTP (ng/ml)	7.3 (2.7–34.3)	3.6 (1.1–50.5)	<0.01	—	—	—
TRACP 5b (mIU/dl)	666 (227–3430)	320 (113–792)	<0.01	882 (310–2858)	293 (147–740)	<0.01
Number of bone BM						
0	0	248	—	0	66	—
1–5	14	—		7	—	
6–19	10	—		5	—	
≧20	10	—		16	—	
Bone metastatic site						
Spine	23	—	—	23	—	—
Pelvis	25	—		25	—	
Others	25	—		23	—	
Tumor volume						
Low	7	—	—	5	—	—
High	27	—		23	—	

Abbreviations: BAP, bone‐specific alkaline phosphatase; BM, bone metastasis; CRP, C‐reactive protein; ECOG‐PS, Eastern Cooperative Oncology Group‐Performance Status; I CTP, carboxy‐terminal pyridinoline cross‐linked telopeptide parts of type I collagen; NTx, cross‐linked N‐terminal telopeptides of type I collagen; PSA, prostate‐specific antigen; tALP, total alkaline phosphatase; TRACP 5b, tartrate‐resistant acid phosphatase type 5b.

**TABLE 2:** The risk factors of bone metastasis in patients with newly diagnosed prostate cancer

	Univariate analysis			Multivariate analysis		
	OR	95% CI	*p* value	OR	95% CI	*p* value
PSA (ng/ml)						
<9.91 versus ≧9.91	20.7	(4.9–88.5)	<0.01	22.6	(5.0–102)	<0.01
tALP (IU/L)						
<218.5 versus ≧218.5	3.2	(1.4–7.1)	0.03	2.6	(0.91–7.22)	0.18
BAP (μg/L)						
<12.2 versus ≧12.2	3.4	(1.5–7.5)	<0.01	1.1	(0.37–3.25)	0.93
NTx (nmol BCE/mmol Cr)						
<13.6 versus ≧13.6	3.8	(1.7–8.8)	<0.01	1.5	(0.57–3.99)	1.0
I CTP (ng/ml)						
<3.8 versus ≧3.8	5.3	(2.2–12.5)	<0.01	3.9	(1.50–9.96)	0.03
TRACP 5b (mIU/dl)						
<335 versus ≧335	21.1	(4.9–89.9)	<0.01	16.6	(3.75–73.8)	<0.01

Abbreviations: BAP, bone‐specific alkaline phosphatase; CI, confidence interval; I CTP, carboxy‐terminal pyridinoline cross‐linked telopeptide parts of type I collagen; NTx, cross‐linked N‐terminal telopeptides of type I collagen; OR, odds ratio; PSA, prostate‐specific antigen; tALP, total alkaline phosphatase; TRACP 5b, tartrate‐resistant acid phosphatase type 5b.

**FIGURE 2:**
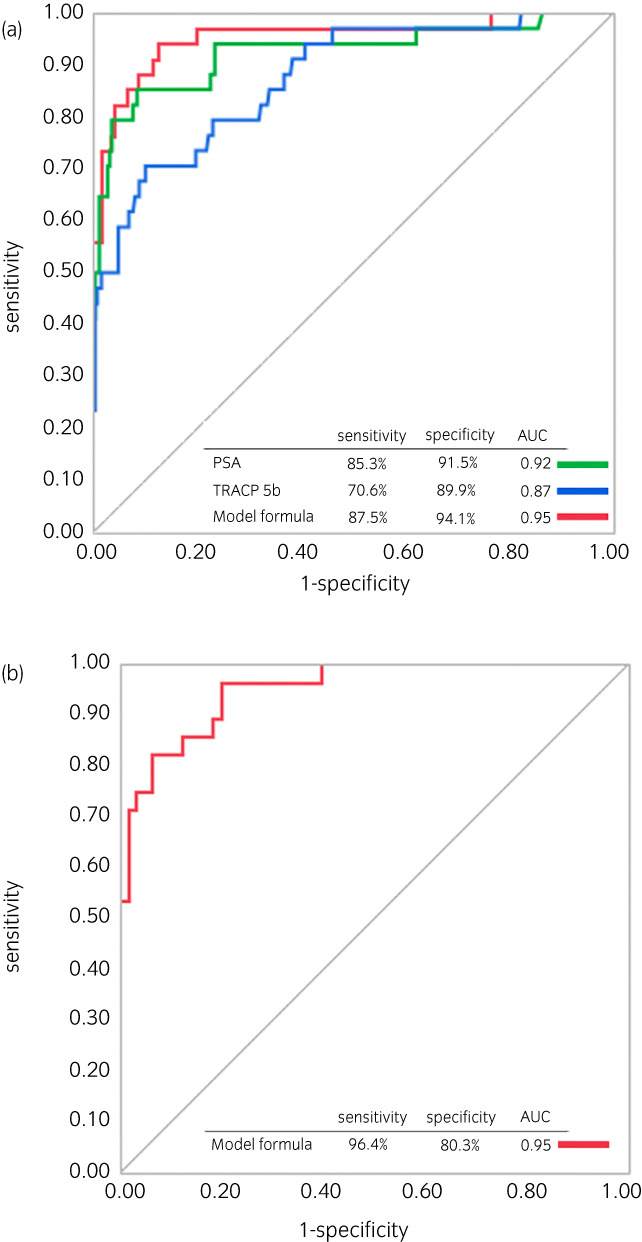
Receiver operating characteristic curves of the predicted probability of bone metastasis by PSA, TRACP 5b, and PSA‐TRACP 5b model formula in discovery cohort (a) and PSA‐TRACP 5b model formula in validation cohort (b). AUC, area under the curve; PSA, prostate‐specific antigen; TRACP 5b, tartrate‐resistant acid phosphatase type 5b.

Next, five bone turnover markers and PSA were examined by stepwise multivariate logistic regression analysis, and TRACP 5b and PSA were chosen as the coefficients for model equation generation, and created BM predictive model by the following formula: *p* = 1/(1 + *e* ^−*x*^), *X* = −5.79 + 0.019 × PSA + 0.0054 × TRACP 5b. The negative predictive value (NPV) of BM was examined in the discovery cohort by dividing the median value of each bone turnover markers and the model formula as cut off values into two groups and NPV of bone turnover markers were high, especially model formula showing 99.1% (Table S1). Using this predictive model, the AUC for detecting BM was 0.95, whereas the AUC for PSA and TRACP 5b were 0.92 and 0.87, respectively (Figure 2a). As the cut‐off value was set so that the sensitivity of BM was greater than 95% (PSA: 7.34 ng/ml, model formula: 0.0404), the sensitivity was 97.1% for PSA alone and 97.1% for the model formula, but the specificity was 37.9% and 80.2%, respectively (*p* < 0.01). When examined by the tumor volume, the sensitivity of the model formula for diagnosis of high‐volume BM was higher than that of low volume BM (*p* < 0.05) (Table S2). Univariate and multivariate analysis revealed that the number of BM (≧20), and *p*‐value of model formula (≧0.0558) independently increased the risk of CSS (*p* < 0.05, respectively) (Table 3).

**TABLE 3:** Univariate and multivariate cox regression analysis predicting CSS and OS in newly diagnosed prostate cancer patients with bone metastasis

	CSS						OS					
	Univariate analysis			Multivariate analysis			Univariate analysis			Multivariate analysis		
	HR	95% CI	*p* value	HR	95% CI	*p* value	HR	95% CI	*p* value	HR	95% CI	*p* value
Age (years)												
<71 versus ≧71	1.4	(0.45–4.8)	0.55	0.9	(0.2–3.1)	0.87	1.6	(0.73–3.7)	0.24	1.2	(0.55–2.8)	0.62
Gleason score												
<8 versus ≧8	19.0	(3.7–349)	<0.01	3.8	(0.39–36.6)	0.19	12.7	(4.4–53.6)	<0.01	7.5	(2.0–28.1)	<0.01
PSA (ng/ml)												
<9.91 versus ≧9.91	13.1	(2.5–239)	<0.01	1.7	(0.16–17.4)	0.66	3.9	(1.6–10.6)	<0.01	1.0	(0.35–3.0)	0.97
Visceral metastasis	10.4	(1.6–40.4)	< 0.01	4.4	(0.73–26.9)	0.14	6.7	(1.6–19.5)	< 0.01	2.7	(0.70–10.7)	0.18
Number of BM												
<20 versus ≧20	48.3	(14.4–169)	<0.01	7.2	(1.2–41.2)	0.039	16.1	(6.2–37.1)	<0.01	5.6	(1.5–20.5)	0.017
CRP (mg/dl)												
<1.0 versus ≧1.0	19.1	(5.1–61.1)	<0.01	2.9	(0.56–15.3)	0.20	6.6	(1.9–17.5)	<0.01	1.5	(0.37–5.9)	0.59
*p* Value of model formula												
<0.0558 versus ≧0.0558	44.7	(8.6–818)	<0.01	9.1	(0.81–101)	0.045	6.4	(2.9–14.5)	<0.01	1.7	(0.58–4.8)	0.36

Abbreviations: BM, bone metastasis; CI, confidence interval; CRP, C‐reactive protein; CSS, cancer‐specific survival; HR, hazard ratio; OS, overall survival; PSA, prostate‐specific antigen.

### Validation cohort

We further enrolled 137 patients in the validation cohort and 94 patients resulted in diagnosis of PCa (Figure 1b) including 28 patients with BM, 5 low tumor volume and 23 high tumor volume (Table 1). Twelve patients had renal dysfunction (12.8%), 9 patients had hepatic dysfunction (9.6%), and no patients had both. Multivariate analysis with TRACP 5b and PSA showed that TRACP 5b (odds ratio [OR] 11.4; 95% confidence interval [CI] 2.1–63.2, *p* < 0.01) and PSA (OR 136; 95% CI 15.0–1226, *p* < 0.01) was an independent useful factor in the diagnosis of BM. The NPV of model formula in validation cohort was 94.6% with above cut off value (TRACP 5b: 335 mIU/dl, combined model: 0.058) (Table S1). AUC of the model equation was 0.95 in validation cohort (Figure 2b). Although the AUC of model formula was higher than that of TRACP 5b alone, it was equivalent to that of PSA alone in the validation cohort (Figure S1). Adapting the above cut off value (PSA: 7.34 ng/ml, combined model: 0.0404) revealed the sensitivity 100%, 96.4% and the specificity of BM 33.3% and 72.7%, respectively, and TRACP 5b increased specificity in this cohort, too (*p* < 0.01).

## DISCUSSION

Approximately, 90% of men with advanced PCa will develop BM.^3^ Recently, approximately 50% of men who convert from androgen‐sensitive state to nonmetastatic castration‐resistant PCa will develop BM within 2 years.^13^ Moreover, the development of systemic therapy has improved the prognosis of PCa patients and increased the occurrence of BM for PCa patients in parallel.^9^ To date, PSA, clinical stage, and Gleason score are known to be the independent predictor of BM.^14^ However, PSA level alone is not sufficient to predict positive findings in bone scintigraphy. Ito et al. reported that 36.1% of patients with PSA < 10 ng/ml had BM in the mass screening populations,^15^ and the other population‐based registry study conversely showed that a fourth of men with PSA > 100 ng/ml did not have distant metastases.^16^ Moreover, the reduction of PSA cutoff value increases the sensitivity but decreases the specificity for diagnosis of BM.^17^ Hence, in this study, we aimed to identify the convenient, noninvasive, and highly specific serum bone turnover markers for the diagnosis of BM and clarified some evidences on the presence of BM in PCa patients.

First, we confirmed that TRACP 5b with cutoff value of 335 mIU/dl was strongly associated with the risk factor of BM in newly diagnosed PCa. TRACP 5b was originally discovered in leukocyte extracts of patients with hairy cell leukemia and later, detected from osteoclasts.^18^ Several studies already reported that bone turnover markers could be the marker of diagnosis of BM in cancers including PCa. However, these markers were measured after the introduction of the hormone therapy, bone modifying agents, and radiation therapy to bone metastatic site,^19^, ^20^ and measured value were easily changeable with these interventions. Moreover, TRACP 5b has high specificity for osteoclasts and is not susceptible to renal and hepatic dysfunction.^18^, ^21^ Indeed, although our discovery and validation cohort included renal or hepatic dysfunction in 61 (21.6%) and 21 (22.3%) patients, respectively, TRACP 5b was useful in the diagnosis of BM in PCa. Considering that urinary bone turnover markers are strongly influenced by renal function and show diurnal variation, serum TRACP 5b is accurate and noninvasive biomarker to predict the presence of BM.^22^, ^23^

Second, we revealed that TRACP 5b and PSA model formula was accurate to detect BM as evidenced by high AUC value (Figure 2) and had high NPV of BM diagnosis (Table S1). Moreover, in multivariate analysis, this formula, not PSA alone, was significantly associated with poor prognosis for CSS in newly diagnosed PCa (Table 3). So far, there are a few reports about the relationship between bone turnover markers such as I CTP, aminoterminal propeptide of type I collagen and BAP and CSS, OS or in newly diagnosed PCa, and included only small number of patients, or crucially excluded an important PCa marker, PSA.^24^, ^25^, ^26^ In contrast, we found that TRACP 5b in accordance with PSA may clearly reflect the presence of BM, and be useful as an adjunct to systemic treatment strategies for PCa with BM. When sensitivity was increased for the purpose of screening, TRACP 5b significantly improved BM diagnostic specificity compared to PSA alone. Given that there are many disadvantages performing bone scintigraphy due to the medical cost and radio exposure,^5^ it might be clinically important to measure TRACP 5b in order to select patients who require bone scintigraphy only when they have a high probability of BM. If bone scintigraphy could be reduced, it would reduce unnecessary exposure and medical costs. C‐reactive protein (CRP) >1.0 mg/dl was a poor prognostic factor for various cancers,^27^ but in this study, only number of BM and model formula were found to be associated with poor prognosis for CSS in multivariate analysis. We revealed that TRACP 5b might be efficient in both diagnosing BM and predicting prognosis in newly diagnosed PCa.

There are some limitations in this study. First, it was a retrospective study and the number of patients was less, and only 12 newly diagnosed PCa patients died of cancer after 61 months of follow‐up, so further large, prospective studies directly comparing the utility of the TRACP 5b‐PSA model equation with current imaging studies are necessary. Second, there was no standardized criteria in determining bone‐modifying agents or anticancer drugs after the hormone therapy, which may affect CSS and OS.

In conclusion, to our knowledge, this is the first study showing a peripheral biomarker, TRACP 5b, as useful tool in diagnosing BM for newly diagnosed PCa. Our data indicate that the model formula using TRACP 5b and PSA was reliable for BM diagnosis and prognosis in newly diagnosed PCa patients and compared to PSA alone, the additional TRACP 5b measurement significantly improved the specificity of BM diagnosis. These findings may allow clinicians determine long‐term treatment strategies for PCa.

## AUTHOR CONTRIBUTIONS

Gaku Yamamichi: Conceptualization; data curation; formal analysis; investigation; writing—original draft; writing—review and editing. Taigo Kato: Conceptualization; formal analysis; investigation; writing—original draft; writing—review and editing. Satoru Yumiba: Conceptualization; data curation; formal analysis; investigation; validation. Eisuke Tomiyama: Conceptualization; data curation; formal analysis; investigation; validation. Yoko Koh: Conceptualization; data curation; formal analysis; investigation; validation. Kosuke Nakano: Conceptualization; data curation; formal analysis; investigation; validation. Makoto Matsushita: Conceptualization; data curation; formal analysis; investigation; validation. Yujiro Hayashi: Conceptualization; data curation; formal analysis; investigation; validation. Yu Ishizuya: Data curation; formal analysis; investigation; validation. Tadashi Watabe: Investigation. Koji Hatano: Conceptualization; data curation; formal analysis; investigation; validation. Atsunari Kawashima: Conceptualization; data curation; formal analysis; investigation; validation. Takeshi Ujike: Conceptualization; data curation; formal analysis; investigation; validation. Yutaka Ono: Data curation; formal analysis; investigation; validation. Tsuyoshi Takada: Data curation; formal analysis; investigation; validation. Shingo Takada: Data curation; formal analysis; investigation; validation. Ryoichi Imamura: Conceptualization; data curation; formal analysis; investigation; validation. Norio Nonomura: Conceptualization; data curation; formal analysis; investigation; validation. Motohide Uemura: Conceptualization; formal analysis; investigation; writing—original draft; writing—review and editing.

## CONFLICT OF INTEREST

None declared.

## APPROVAL OF THE RESEARCH PROTOCOL BY AN INSTITUTIONAL REVIEWER BOARD

This study was approved by the Institutional Review Board of Osaka University Hospital (# 13397‐19).

## INFORMED CONSENT

Informed consent was obtained from all individual participants.

## REGISTRY AND THE REGISTRATION NO. OF THE STUDY/TRIAL

N/A.

## ANIMAL STUDIES

N/A.

## Supporting information


Figure S1.

Table S1
