# Supplementary figures and images for: Diagnostic and prognostic significance of tartrate‐resistant acid phosphatase type 5b in newly diagnosed prostate cancer with bone metastasis: A real‐world multi‐institutional study
Source: Int J Urol. 2022 Oct 28;30(1):70–6. doi: 10.1111/iju.15063 (PMC10092858; doi:10.1111/iju.15063)

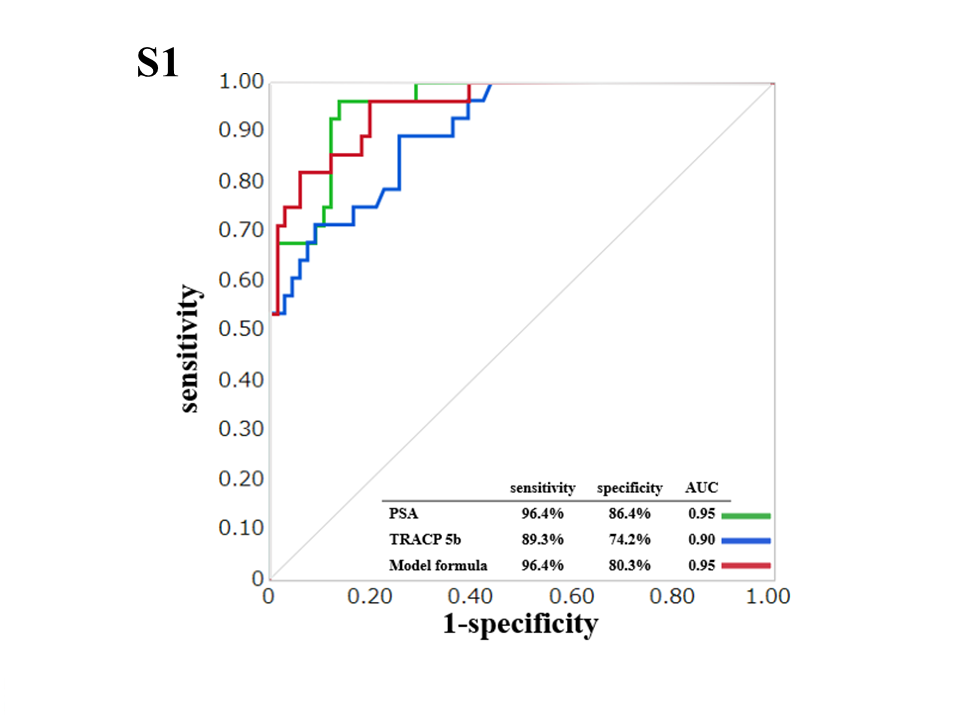

Supplement: Supplementary file 1 — Figure S1. [file IJU-30-70-s002.tif]
