# Supplementary material for: Diagnostic and prognostic significance of tartrate‐resistant acid phosphatase type 5b in newly diagnosed prostate cancer with bone metastasis: A real‐world multi‐institutional study
Source: Int J Urol. 2022 Oct 28;30(1):70–6. doi: 10.1111/iju.15063 (PMC10092858; doi:10.1111/iju.15063)
Supplement: Supplementary file 2 — Table S1 [file IJU-30-70-s001.docx]

| **Table S1** Negative predictive value of bone metastasis | | |
| --- | --- | --- |
|  | Discovery cohort | Validation cohort |
|  | NPV | NPV |
| tALP | 93.6% (132/141) | - |
| BAP | 93.8% (136/145) | - |
| NTx | 94.4% (134/142) | - |
| I CTP | 95.3% (143/150) | - |
| TRACP 5b | 98.6% (141/143) | 93.8% (45/48) |
| Model formula | 99.1% (217/219) | 94.6% (53/56) |
| *tALP* total alkaline phosphatase, *BAP* bone specific alkaline phosphatase, *NTx* cross-linked N-terminal telopeptides of type I collagen, *I CTP* carboxy-terminal pyridinoline cross-linked telopeptide parts of type I collagen, *TRACP 5b* tartrate-resistant acid phosphatase type 5b, *NPV* negative predictive value | | |

| **Table S2** Sensitivity and specificity of model formula for diagnosis of bone metastasis classified by tumor volume | | | | |
| --- | --- | --- | --- | --- |
|  | Discovery cohort | | Validation cohort | |
|  | Sensitivity | Specificity | Sensitivity | Specificity |
| Low volume | 71.4% (5/7) | 87.5% (217/248) | 60.0% (3/5) | 80.3% (53/66) |
| High volume | 100% (27/27) | 87.5% (217/248) | 95.7% (22/23) | 80.3% (53/66) |
| P value | 0.037 | - | 0.073 | - |
|  | | | | |
